# The 2022 focused update of the 2018 Korean Hypertension Society Guidelines for the management of hypertension

**DOI:** 10.1186/s40885-023-00234-9

**Published:** 2023-02-15

**Authors:** Hack-Lyoung Kim, Eun Mi Lee, Shin Young Ahn, Kwang-il Kim, Hyeon Chang Kim, Ju Han Kim, Hae-Young Lee, Jang Hoon Lee, Jong-Moo Park, Eun Joo Cho, Sungha Park, Jinho Shin, Young-Kwon Kim

**Affiliations:** 1 Department of Internal Medicine, Seoul Metropolitan Government Seoul National University Boramae Medical Center, Seoul National University College of Medicine, Seoul, Republic of Korea; 2 Department of Internal Medicine, Wonkwang University Sanbon Hospital, Wonkwang University School of Medicine, Gunpo, Republic of Korea; 3 Department of Internal Medicine, Korea University Guro Hospital, Korea University College of Medicine, Seoul, Republic of Korea; 4 Department of Internal Medicine, Seoul National University Bundang Hospital, Seoul National University College of Medicine, Seongnam, Republic of Korea; 5 Department of Preventive Medicine, Yonsei University College of Medicine, Seoul, Republic of Korea; 6 Department of Internal Medicine, Chonnam National University Hospital, Gwangju, Republic of Korea; 7 Department of Internal Medicine, Seoul National University Hospital, Seoul National University College of Medicine, Seoul, Republic of Korea; 8 Department of Internal Medicine, Kyungpook National University Hospital, Kyungpook National University School of Medicine, Daegu, Republic of Korea; 9 Department of Neurology, Uijeongbu Eulji Medical Center, Eulji University, Uijeongbu, Republic of Korea; 10 Department of Internal Medicine, Yeouido St. Mary’s Hospital, College of Medicine, The Catholic University of Korea, Seoul, Republic of Korea; 11 Department of Internal Medicine, Severance Hospital, Yonsei University College of Medicine, Seoul, Republic of Korea; 12 Department of Internal Medicine, Hanyang University Medical Center, Hanyang University College of Medicine, Seoul, Republic of Korea; 13 Department of Internal Medicine, Dongguk University Ilsan Hospital, Dongguk University School of Medicine, Seoul, Republic of Korea

**Keywords:** Blood pressure, Guideline, Hypertension, Korea

## Abstract

Hypertension is the leading cause of death in human being, which shows high prevalence and associated complications that increase the mortality and morbidity. Controlling blood pressure (BP) is very important because it is well known that lowering high BP effectively improves patients’ prognosis. This review aims to provide a focused update of the 2018 Korean Hypertension Society Guidelines for the management of hypertension. The importance of ambulatory BP and home BP monitoring was further emphasized not only for the diagnosis but also for treatment target. By adopting corresponding BPs, the updated guideline recommended out-of-office BP targets for both standard and intensive treatment. Based on the consensus on corresponding BPs and Systolic Blood Pressure Intervention Trial (SPRINT) revisit, the updated guidelines recommended target BP in high-risk patients below 130/80 mmHg and it applies to hypertensive patients with three or more additional cardiovascular risk factors, one or more risk factors with diabetes, or hypertensive patients with subclinical organ damages, coronary or vascular diseases, heart failure, chronic kidney disease with proteinuria, and cerebral lacunar infarction. Cerebral infarction and chronic kidney disease are also high-risk factors for cardiovascular disease. However, due to lack of evidence, the target BP was generally determined at < 140/90 mmHg in patients with those conditions as well as in the elderly. Updated contents regarding the management of hypertension in special situations are also discussed.

## Level of evidence and classes of recommendations

Table 1 shows the levels of evidence and classes of recommendations defined in these updated guidelines.

**Table 1:** Definition of the level of evidence and class of recommendation

Criteria	Definition
Level of evidence	
A	Data derived from multiple randomized controlled trials or meta-analyses.
B	Data derived from a single randomized controlled trial or nonrandomized clinical trials.
C	Experts’ opinion or data derived from limited evidence.
Class of recommendation	
I	Evidence and/or general agreement that a given treatment or procedure is beneficial, useful, and effective. Therefore, it is recommended.
IIa	Conflicting evidence and/or a divergence of opinion about the usefulness/efficacy of the given treatment or procedure exists. However, in general, weight of evidence/opinion is in favor of usefulness/efficacy. Therefore, it is reasonable to be performed.
IIb	Usefulness/efficacy less well established by evidence/opinion. Therefore, it may be considered.
III	Evidence or general agreement that a given treatment or procedure is not beneficial and may be harmful in some case. Therefore, it is not recommended.

## Clinical evaluation of hypertension

### Blood pressure measurement

Accurate measurement of blood pressure (BP) is essential for the diagnosis, management, and risk stratification of hypertension. Measured BP varies according to the measurement environment, behavior of individuals, measurement protocol, device used for the measurement, and technical skill of observers. Therefore, the diagnosis of hypertension should be confirmed by repeat office BP (OBP) measurements with standardized methods at repeat office visits (Table 2) [1, 2]. Also, out-of-office BP measurement with either ambulatory BP monitoring (ABPM), or home BP monitoring (HBPM) is recommended as a complementary strategy for the diagnosis of hypertension [3].

**Table 2:** Standardized BP measurement

Process	Recommendation
Proper preparation	Resting for 5 min in a quiet room.
	No smoking, alcohol, or caffeine for 30 min before measurement.
	No talking by individual or observer during measurement or between measurements.
	Emptying bladder before measurement.
Proper posture	Sitting in a chair with back support.
	Legs uncrossed and feet kept flat on the floor.
	Bare upper arm or upper arm with light clothes resting on the table.
Proper technique	
Use of validated device	Auscultatory device or automated device.
Use the correct cuff size	
Auscultatory device	Inflatable bladder length which is 75–100% of an individual’s middle upper-arm circumference and width 37–50% of the arm circumference.
Automated device	Select cuff size according to the device’s instructions.
Placement of cuff at the heart level	The middle portion of the cuff on the individual’s upper arm at the mid-sternal level (lower end of the cuff 2–3 cm above the antecubital fossa)
Measurement with auscultatory device	Estimate radial pulse obliteration pressure and inflate the cuff 20–30 mmHg above this level for auscultatory determination of the BP level.
	Place the stethoscope (bell side) on the brachial artery at the point of maximal pulsation.
	For auscultatory readings, deflate the cuff pressure 2 mmHg per beat or second and listen to Korotkoff sounds.
	Document accurate BP readings properly: Record SBP as the onset of first Korotkoff sound (K1) and diastolic DBP as disappearance of all Korotkoff sound (K5). Record as DBP at the fourth Korotkoff sound (K4) in pregnancy, arteriovenous shunt, and chronic aortic insufficiency.
Repeated measurements	Separate repeated measurements in intervals of 1–2 min.
BP measurement in both arms	Measure BP in both arms at the first visit and then the arm with the higher BP should be used at subsequent visits.
Positional BP measurement	Measure BP 1 and 3 minutes after standing from a seated position in older people, people with diabetes, and people with suspected orthostatic hypotension.
BP measurement in arrhythmia	Take triplicate BP measurements and use their average.
Pulse measurement	Record heart rate and use pulse palpation at rest to exclude arrhythmia.
BP measurement in the leg	Measure leg BP in suspected peripheral arterial disease if the lower extremity pulse is weak.
	Measure ankle BP, in the supine position using a validated automated device with the cuff placed around the ankle/lower calf.

*BP* blood pressure, *SBP* systolic blood pressure, *DBP* diastolic blood pressure

#### Office blood pressure measurement

**Table:** 

Recommendation	Class	Level	Reference
Repeated OBP with standardized methods is recommended at repeat office visits for the diagnosis of hypertension.	I	C	[1, 2]
OBP measurement with reliable non-mercury sphygmomanometers is recommended.	I	A	[4]

Mercury sphygmomanometers are banned in Korea in terms of Minamata Convention for the environmental concern of mercury; thus, BP should be measured with a validated non-mercury sphygmomanometer (dabl, Dublin, Ireland; http://www.dableducational.org) [2]. They are divided into two types of devices according to measurement techniques: auscultatory (Korotkoff method) and oscillometric devices (automated electronic device) [2]. Among them, auscultatory devices include aneroids and hybrid sphygmomanometers. Aneroids (mechanical type with a dial) are commonly used in clinical practice, but they require regular calibration to ensure the accuracy of the devices. Hybrid sphygmomanometers replace the mercury column with an electronic pressure column (manual electronic auscultatory device). BP measurement techniques and reading skills of the scale of these devices are similar to those of mercury sphygmomanometers, but they reduce the impact of observer error because meniscus sign is negligible [2, 4]. In oscillometric devices, mean arterial BP is estimated to be cuff pressure when oscillation amplitude is maximal, and then systolic BP (SBP) and diastolic BP (DBP) are computed by proprietary algorithms that are known only to the manufacturer. Therefore, since different devices are not interchangeable, these devices should be validated separately according to an established protocol [2]. These devices may be inaccurate in some populations including old ages, pregnant women, children, individuals with very large or thin arms, and patients with arrhythmias or stiffened arteries [2, 4].

To obtain accurate BP reading, reliable upper-arm cuff devices and proper techniques should be used (Table 2). BP is measured siting in a chair with back support with the upper arm at the heart level 5 minutes after rest. A cuff placement below the heart level leads to an overestimation of BP. Cuff to fit arm size is selected. A cuff smaller than required overestimates BP. For auscultatory devices, inflatable bladder length which is 75 to 100% of the individual’s middle upper-arm circumference and width 37 to 50% of the arm circumference is used. In automated devices, cuff size is selected according to the device’s instructions [2]. For auscultatory readings, deflate cuff pressure 2 mmHg per beat or second. Faster deflation can lead to an underestimation of SBP and overestimation of DBP. At least two measurements of BP are taken and the average of the readings is used. BP is measured in both arms at the initial visit. Interarm SBP difference of > 10 mmHg must be confirmed with repeated measurements and then the arm with the higher BP should be used at subsequent visits. Persistent interarm SBP difference of above 20 mmHg is considered the presence of arterial diseases such as coarctation of aorta or upper-extremity arterial obstruction [5]. If upper-arm BP cannot be measured due to a malformation or an arterial stenosis, then it should be measured in the legs. If the pulses of the lower extremities are weak, BP should be measured in the legs to exclude the presence of peripheral arterial disease. There is no consensus on measuring BP in the legs [6]. Ankle BP, rather than calf or thigh BP, is measured in a supine position using a validated automated device with the cuff placed around the ankle/lower calf because where the cuff generally causes less discomfort and is easier to fit. Auscultatory technique is not feasible in most subjects; thus, it is not recommended to measure BP in the legs. Triplicate BP readings and the average of readings are required in patients with arrhythmias (particularly atrial fibrillation) because of beat-to-beat changes in BP [7]. Positional BP measurement is recommended in diabetes mellitus (DM), old ages, and suspected orthostatic hypotension. All the hypertensive patients undergo pulse palpation at rest to determine heart rate and search for arrhythmias such as atrial fibrillation [8, 9].

#### Out-of-office blood pressure measurement

Since BP is influenced by many factors, including environment, emotion, and circadian changes, a single office BP measurement can lead to incorrect diagnosis and unnecessary treatment for hypertension. Therefore, repeated measurements of OBP as well as out-of-office BP measurements of ABPM and HBPM are needed for proper diagnosis and treatment of hypertension. Among out-of-office BP measurements, ABPM is first recommended because it provides more comprehensive information on BP such as BP phenotypes, circadian BP patterns, and BP variability [10–12]. However, HBPM is often a more practical in usual environment for its ease to use [13–15]. Out-of-office BP measurement provides better prognostic information than OBP measurement alone [16–19].

##### Ambulatory blood pressure monitoring

**Table:** 

Recommendation	Class	Level	Reference
ABPM is recommended for a number of clinical indications, such as diagnosing hypertension, identifying white coat hypertension (WCH) and masked hypertension (MH), quantifying the effects of treatment, and estimating prognosis.	I	A	[3, 16, 20, 21]

The main benefit of ABPM is to provide average BP readings of daytime, nighttime, and 24-hours that cannot be detect by OBP alone [10–12]. Thus, ABPM provides information on BP phenotypes (WCH, MH, and sustained hypertension), circadian patterns (dipper, nondipper, reverse dipper, extreme dipper, and morning surge), and BP variability. Most of the ABPM devices are automated and programmable to measure BP by validated oscillometric devices. To obtain satisfactory ABPM data, nondominant upper arm with a proper cuff should be used. BP readings are obtained every 15 minutes to 30 minutes during the daytime and every 30 minutes to 60 minutes during the nighttime. Appropriate ABPM provides at least 20 awake and 7 asleep BP readings, of which 70% should be valid [12]. When measuring ABPM, it is important to instruct individuals to keep usual daily activities and nighttime sleep and to avoid strenuous exercise. They are asked to keep the body, especially their arm, still during each BP measurement. It is also necessary to instruct them to write in a diary. The key indications for ABPM are summarized in Table 3. ABPM is indicated in clinical conditions such as (1) confirmation of hypertension, (2) detection of WCH, MH, and dipping patterns, (3) assessment of the BP variability, and (4) monitoring antihypertensive medication efficacy in treated individuals [2, 11, 12]. ABPM thresholds for hypertension is ≥130/80 mmHg over 24 hours, ≥135/85 mmHg for the daytime average, and ≥ 120/70 mmHg for nighttime average (all equivalent to OBP ≥140/90 mmHg) (Table 4) [11]. Physiologically, BP decreases during sleep. If nocturnal BP decreases by 10 to 20% compared to daytime levels, it is called dipper hypertension. However, dipping status is poorly reproducible [22]. ABPM provides more information on hypertension-mediated organ damage (HMOD) than OBP. If nocturnal BP has a less than 10% fall (nondipper), it is associated with high risk of left ventricular hypertrophy, myocardial ischemia, and death compared to dipper hypertension [23–25]. Reverse dippers may also have autonomic dysfunction [26] and be at high risk of hemorrhagic stroke and cardiovascular (CV) mortality [27, 28]. BP decrease of 20% or more (extreme dipper) may be associated with high risk for ischemic stroke and atherosclerosis [27]. A morning surge is considered a risk factor for CV disease (CVD), particularly stroke [29]. However, definition, reproducibility, and treatment of the morning surge should be answered [30].

**Table 3:** Indications for ambulatory BP monitoring

Confirm the diagnosis of hypertension.	
Detect the white coat hypertension.	
Detect the masked hypertension in individuals with high-normal OBP or normal BP with target organ damage or high cardiovascular risk.	
Detect the hypertension in cases of marked BP discrepancy between OBP and home BP.	
Assess the dipping patterns (dipper, nondipper, reverse dipper, and extreme dipper), nocturnal hypertension, morning hypertension, and morning surge.	
Assess the cause of secondary hypertension (e.g., sleep apnea).	
Assess labile hypertension or hypotension (postural, postprandial, and drug-induced hypotension).	
Assess the BP caused by autonomic dysfunction.	
Assess the short-term BP variability.	
Monitoring the efficacy of antihypertensive medications in treated patients.	
Assess the white coat effect and masked uncontrolled hypertension.	
Assess symptomatic hypotension due to excessive treatment.	
Ensure 24-hour BP control (particularly in high-risk individuals and pregnant women).	
Confirm the diagnosis of resistant hypertension.	
Assess accurate BP measurement for risk assessment.	

*BP* blood pressure, *OBP* office blood pressure

**Table 4:** Threshold for diagnosing hypertension

Category	Systolic BP (mmHg)	Diastolic BP (mmHg)
Office BP	≥140	≥90
Ambulatory BP		
24 Hours	≥130	≥80
Daytime	≥135	≥85
Nighttime	≥120	≥70
Home BP	≥135	≥85

*BP* blood pressure

##### Home blood pressure monitoring

**Table:** 

Recommendation	Class	Level	Reference
HBPM is recommended for the diagnosis of hypertension, detection of WCH, MH, and sustained hypertension, and estimation of prognosis.	I	A	[3, 13, 18]
It is recommended that all individuals should be instructed about proper BP measurements.	I	C	[15, 31]

HBPM has the main advantages of easy to use, relatively low cost, and long-term BP monitoring in treated individuals with hypertension [13–15]. Thus, HBPM can be implanted during usual daily activities. However, BP measurement during HBPM is mostly unattended without medical supervision; thus, standardized protocol should be provided to all individuals for accurate readings (http://www.koreanhypertension.org/sense/family) (Table 5). Finger devices are not used due to inconsistent measurements. Wrist devices are generally not used due to their low accuracy when not positioned at the heart level; thus, validated wrist devices can be used in people with very large arms when upper-arm cuffs are not available. Automated upper-arm devices are currently validated and approved [2, 13, 15]. BP is measured 5 minutes after rest in a sitting position. However, considering the efficiency of BP measurement, a shorter rest of 2 minutes may be an alternative to a rest of 5 minutes before BP measurement [32]. A cuff to fit the arm is selected according to the device’s instructions, which is placed at the heart level. Accurate HBPM for accuracy is summarized in Table 5. HBPM is employed to confirm the diagnosis of hypertension, and to detect WCH, MH, and resistant hypertension. There is accumulating evidence that HBPM with or without telemonitoring may improve medication adherence in treated individuals with hypertension [33–35]. In additions, HBPM is cost-effective in diagnosing and treating hypertension [36], and better predicts for HMOD and CV morbidity and mortality than OBP [18, 19]. However, HBPM is often inaccurate in arrhythmias, old ages, or pregnancy. Also, since HBPM gives little or no information on BP at work or during sleep, it does not help assess nocturnal hypertension, extreme BP decrease during sleep, and morning surge [37]. Compared to OBP readings, HBPM values are usually lower; thus, HBPM threshold for diagnosing hypertension is ≥135/85 mmHg (equivalent to OBP ≥140/90 mmHg) in Table 4 [2, 14, 15].

**Table 5:** Home BP measurement

Process	Recommendation
Preparation	Resting for 5 min in a quiet room with comfortable temperature.
	No smoking, alcohol or caffeine, exercise, and bathing 30 min before measurement.
	No talking during measurement and between measurements.
Position	Sitting in a chair with back support.
	Legs uncrossed and feet kept flat on the floor.
	Upper arm resting on the table with wearing light clothes.
Technique	
Device	Validated automated upper-arm device.
Cuff	Select proper cuff size according to the device’s instructions
Proper placement of cuff at the heart level	Placement of cuff on the mid-arm with the lower edge of the cuff 2–3 cm above the antecubital fossa.
Measurement time	
Morning	Before a meal and after urination, and before drug intake if treated.
Evening	1 Hr before sleep.
Measurement frequency	Two measurements with 1-min intervals.
Measurement schedule	Measurement for at least 5 day, especially for 7 day for first diagnosing hypertension (discard 1st day readings and use their average values)
Recording	Record all readings in BP log accurately All BP recordings in the built-in memory of device should be brought to clinic appointments BP values should not be selectively recorded by individual.

*BP* blood pressure

#### White coat hypertension and masked hypertension

**Table:** 

Recommendation	Class	Level	Reference
Out-of-office BP with either ABPM or HBPM is recommended to detect WCH.	I	A	[10, 13]
In individuals with WCH, regular BP monitoring with either ABPM or HBPM is reasonable to detect transition to sustained hypertension.	IIa	B	[38, 39]
In treated individuals with uncontrolled OBP, out-of-office BP with either ABPM, or HBPM is reasonable to detect white coat effect (WCE).	IIa	C	[40, 41]
In individuals with high-normal OBP or normal OBP accompanying target organ damage, screening for MH with ABPM or HBPM may be considered.	IIb	B	[20, 21]
In individuals with high-normal OBP or normal OBP accompanying target organ damage, screening for masked uncontrolled hypertension (MUCH) with ABPM or HBPM may be considered.	IIb	C	[21]

According to results of OBP and out-of-office measurements, individuals are categorized into four phenotypes: (1) normotension (OBP and out-of-office BP not elevated), (2) WCH (elevated OBP [≥140/90 mmHg] but not elevated out-of-office BP [≤135/85 mmHg in awake BP and home BP or ≤ 130/80 mmHg in 24-hour mean BP]) [11, 42], (3) MH (elevated out-of-office BP but not OBP) [43], and (4) sustained hypertension (both elevated OBP and out-of-office BP). Hypertension with WCE or white coat uncontrolled hypertension (WCUH) describes elevated OBP but not HBPM or ABPM in treated individuals [11]. MUCH describes elevated out-of-office BP but not OBP in treated individuals [11].

##### White coat hypertension

Although different among studies, the prevalence of WCH is approximately 15 to 30% in the general population [20, 42, 44] and hypertension with WCE or WCUH is 30 to 40% in treated individuals [40, 41]. According to the registry data on ABPM in secondary or tertiary centers supported by the Korean Society of Hypertension (Korean Ambulatory Blood Pressure Monitoring [Kor-ABP] Registry), WCH was estimated to be 14.9% of 1916 individuals who underwent ABPM for the diagnosis of hypertension and WCUH was 13.5% of all treated individuals [45]. WCH occurs more frequently in female sex, old age, no smoking, pregnancy, low body mass index, and stage I hypertension [42, 46–48] but not in patients with HMOD [45]. WCH confers lower short-term risk within 5 years but is associated with higher CV events [49] and progress to sustained hypertension during long-term follow-up [38, 39]. Thus, patients with WCH believe that periodic BP monitoring can detect transition to sustained hypertension. WCUH does not predict CV morbidity and mortality [50, 51]; however, the clinical implication is that it might lead to uncontrolled or pseudoresistant hypertension [41]. Thus, further assessment of BP by ABPM or HBPM should be considered before starting medication or dose escalation in suspected WCH or WCUH.

##### Masked hypertension

MH occurs approximately 9 to 30% in individuals with normal OBP [52, 53] and MUCH occurs approximately 30 to 60% of treated individuals [48, 50]. According to the Kor-ABP Registry data, MH was observed in 17.6% of individuals who underwent ABPM for the diagnosis of hypertension, and MUCH was also observed in 13.8% of patients taking antihypertensive medications and in 35.1% of individuals with controlled OBP [45, 54]. Young age, male sex, cigarette smoking, alcohol drinking, anxiety, job stress, higher level of physical activity, DM, chronic kidney disease (CKD), and obstructive sleep apnea are associated with MH [55]. In Kor-ABP Registry data, when hypertension was defined by mean 24-hour BP ≥130/80 mmHg, MUCH is associated with high-normal OBP, underuse of antihypertensive drugs, dyslipidemia, prior stroke, and left ventricular hypertrophy [54]. MH may progress to sustained hypertension [38] and both MH and MUCH have been associated with HMOD [20, 21]. There are still limited data on the threshold or reproducibility of MH and MUCH diagnoses [56, 57]. However, in individuals with normal or high-normal BP, screening for MH or MUCH suing ABPM or HBPM may be considered if they were smokers or alcoholics, or have metabolic syndrome, target organ damage, DM, exercise induced hypertension, or high job stress.

#### Unattended automated office blood pressure measurement

Unattended automated OBP (AOBP) is measured three times at 1-minute intervals after 5-minute rest in a quiet environment without any medical staff using an automated device [58]. However, the protocol for measurement frequency and interval, and rest duration is inconsistent among studies [59]. Although AOBP may reduce WCE [59], MH can be detected using AOBP as usual OBP [60]. The BP readings using unattended AOBP give lower than usual OBP values; thus, threshold for diagnosing hypertension is similar to awake ambulatory BP [61]. However, patients with mean office SBP < 130 mmHg generally have lower OBP values than the corresponding awake ambulatory BP, regardless of their treatment status [62].

#### Blood pressure measurement using cuffless wearable devices

Several novel cuffless wearable mobile devices, such as smartwatches or smartphones, are used for BP measurement [2, 63]. They have sensors, which evaluate the pulsation of arterioles and estimate BP based on pulse wave velocity or other technologies. Recently, BP measurement using a photoplethysmography-based smartphone algorithm paired with a smartwatch is approved as a medical device, fulfilling International Organization for Standardization (ISO) standards. These devices have great benefits because they can easily and conveniently obtain multiple or even continuous BP measurements for days or weeks and there is no cuff-induced measurement error. However, they have two critical limitations that accuracy of sensor-based BP measurement and the reliability of wrist BP devices are low. BP values differ according to measurement methods; thus, they are presented as BP ranges rather than actual BP values. Future clinical practice requires device validation with an established protocol, BP measurement with standardization, education of BP measurement, and regular calibration with a reference device.

#### Central blood pressure measurement

Approximately 80% of brachial arterial pressure may be attributed to central arterial pressure. In addition, central BP has been reported to more closely correlate with target organ damage or prognosis [64]. Although central BP might be useful for patients with significant differences between central and brachial artery pressures, it is not superior to conventional brachial BP in the treatment of hypertension [65].

#### Hypertension screening

**Table:** 

Recommendation	Class	Level	Reference
Hypertension screening using standard BP measurements is recommended for all adults aged ≥20 years.	I	B	

Because it is well known that the higher the BP, the higher the risk of CVD and death [66, 67], regular screening tests are strongly recommended to determine the presence of hypertension in all adults, except those who have already been diagnosed with hypertension. Available evidence on optimal screening intervals for the detection of hypertension remains limited [68].

**Fig. 1:**
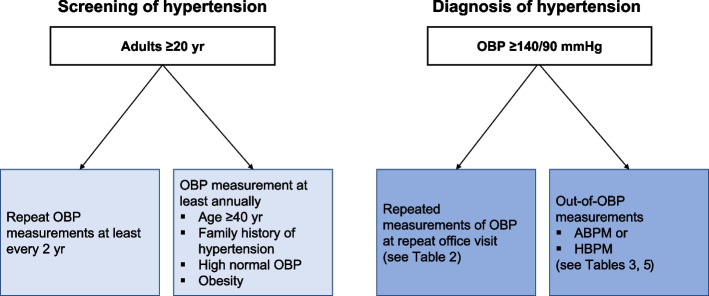
Screening and diagnosis of hypertension. OBP, office blood pressure; HBPM, home blood pressure monitoring; ABPM, ambulatory blood pressure monitoring

However, many observational studies have shown that the earlier the hypertension is detected and treated, the lower the risk of CVD [69, 70], which is indirect evidence for the benefit of early diagnosis of hypertension through screening in adults. In a randomized clinical trial of elderly community people in Canada, hospitalizations for CVD within 1 year were reduced by 9% in the group that received pharmacy-based hypertension screening and risk assessment compared to the group that did not [71]. Previous studies have reported neither serious side effects or increased risk of disease associated with screening for hypertension, nor its negative effects on quality of life or psychological status [72–74]. Although ABPM can cause minor side effects, such as sleep disturbance, pain/anxiety, bruising, and skin irritation [75–77], it may not be problematic because primary screening for hypertension is based on OBP. There is insufficient evidence on the optimal frequency of screening for hypertension in adults. BP measurement does not have serious side effects and the cost is not high, in many countries, the hypertension screening cycle is determined by considering the medical environment or health examination infrastructure level of their own countries rather than the result of cost-effectiveness analysis [68, 78]. The Korean Society of Hypertension suggests regular OBP measurement based on health checkups by the National Health Insurance Service in Korea. All adults who are over 20 years old are recommended for OBP measurements at least every 2 years. In adults aged ≥40 years, with a family history of hypertension, high-normal OBP (130–139/80–89 mmHg), or obesity, OBP measurement are indicated at least annually because of the high rate of progression of hypertension (Fig. 1).

#### Corresponding blood pressure

It is recommended to apply the same corresponding BP to the diagnosis and treatment of hypertension (Table 6). It is very similar to the 2017 American guidelines provided so that the concern of WCE and overtreatment needs to be balanced with the risk of mask effect and undertreatment during intensive BP control targeting BP below 130/80 mmHg.

**Table 6:** Corresponding BP

	Office BP	Ambulatory BP (24 hours)	Ambulatory BP (daytime)	Home BP
Systolic BP (mmHg)	140	130	135	135
Systolic BP (mmHg)	130	125	130	130

*BP* blood pressure

### Laboratory examination

**Table:** 

Recommendation	Class	Level	Reference
It is reasonable that the routine laboratory tests should be evaluated at the first visit and annually.	IIa	C	

Laboratory examinations are performed to identify additional CV risk factors, secondary causes of hypertension, subclinical organ damage, and concomitant diseases. Routine laboratory tests are mandatory before antihypertensive treatment. Other recommended and extended tests can be performed if necessary (Table 7). In the 12-lead electrocardiogram, the findings of left ventricular hypertrophy, left bundle branch block, and myocardial infarction are regarded as high risk for CVD. Proteinuria or hematuria suggests CKD, and glycosuria suggests DM. Blood hemoglobin and hematocrit levels can determine anemia. An increase in the volume of red blood cells is related to an increase in BP, but its correlation coefficient is very low. If hypokalemia is observed in the baseline evaluation, it suggests excessive state of inorganic corticoids such as primary aldosteronism as a cause of hypertension. In addition, baseline potassium levels should be evaluated because thiazide or loop diuretics can lose serum potassium. Hypokalemia is associated with increased lethargy, arrhythmias, and the incidence of DM and hyperkalemia can be caused by impaired renal function. An increase in serum creatinine level or a decrease in the estimated glomerular filtration rate (eGFR; < 60 mL/min/1.73m^2^) indicates a decrease in kidney function [79]. When eGFR based on serum creatinine is inaccurate, it is recommended to measure serum cystatin C and eGFR using cystatin C in combination with serum creatinine. The serum concentration of cystatin C is not associated with sex, age, or muscle mass. Therefore, cystatin C is useful for the diagnosis of renal impairment in young men with high muscle mass and elderly women with low muscle mass [80–82]. An increase in uric acid level is observed in gout, impaired renal function, obesity, or diuretic use. Fasting blood glucose and lipid tests are necessary for confirming hyperglycemia and dyslipidemia, respectively. In addition, when diuretics or β-blockers have long been used, the incidence of hyperglycemia and dyslipidemia increases, which can be evaluated by routine tests. Thyroid stimulating hormone is a useful indicator to confirm hypothyroidism and hyperthyroidism. An increase in cardiac to thoracic ratio on chest X-ray or pulmonary congestion/edema suggests heart failure. Calcification of the aortic arch represents arteriosclerosis. Proteinuria can be evaluated using a urine reagent strip, urine protein to creatinine ratio, or urine albumin to creatinine ratio (ACR). Considering the sensitivity and accuracy of the tests, the urine ACR is recommended. The first morning urine specimen is preferred for the detection of albuminuria. However, a random urine sample is acceptable if no first morning urine specimen is available. Albuminuria can be transiently observed in several pathologic and physiologic conditions such as, urinary tract infection, excessive exercise, taking nonsteroidal anti-inflammatory drugs, and menstrual blood in women. Albuminuria is defined as a urine ACR ≥30 mg/g (≥3 mg/mmol), and can be confirmed when the urine ACR > 300 mg/g (> 30 mg/mmol). If a urine ACR ≥30 mg/g (≥3 mg/mmol) is detected on no less than two occasions over at least 3 months, persistent albuminuria as a marker of kidney damage is present [83, 84]. It is recommended that routine laboratory tests should be evaluated at the first visit and annually. If the degree of hypertension is severe or if the hypertension is not well controlled even with standardized drug treatment, additional tests can be performed to evaluate asymptomatic organ damage or if clinically necessary. Transthoracic echocardiography is useful for diagnosing left ventricular hypertrophy. The measurement of carotid intima-media thickness is not recommended because it is not standardized and the clinical evidence is weak. Identification of carotid atherosclerotic plaques can be helpful in predicting prognosis.

**Table 7:** Laboratory examination

Test	Examination
Routine test	12-Lead electrocardiogram
	Urinalysis (proteinuria, hematuria, and glucosuria)
	Hemoglobin, hematocrit
	K+, creatinine, eGFR^a)^, and uric acid
	Fasting glucose, lipids (total cholesterol, high-density lipoprotein-cholesterol, low-density lipoprotein-cholesterol, and triglyceride)
	Chest X-ray
	Microalbuminuria^b)^ (albumin/creatinine [a random urine sample])
Recommended test	75 g oral glucose tolerance test or hemoglobin A1c (if fasting glucose ≥100 mg/dL)
	Transthoracic echocardiography
	Carotid ultrasound (plaque)
	Ankle-brachial blood pressure index
	Pulse wave velocity
	Fundoscopy (mandatory in diabetes)
	24-hr urine protein excretion
	Cystatin C^c)^
Extended test	Search for subclinical organ damage (brain, heart, kidney, and vessels)
	Search for secondary causes of hypertension

eGFR, estimated glomerular filtration rate

^a)^By CKD-EPI (Chronic Kidney Disease Epidemiology Collaboration) equation; ^b)^eGFR < 60 mL/min/1.73m^2^, a follow-up interval of 3 to 6 months is recommended; ^c)^Useful for evaluating renal function in younger men with high muscle mass or older women with low muscle mass

## Treatment of hypertension

### Cardiovascular risk and treatment plan

The CV risk and treatment strategy for hypertension is shown in Table 8. Antihypertensive medication is generally not recommended for patients with prehypertension due to limited evidence [85]. Patients with stage 1 hypertension with low CV risk need antihypertensive medications according to their BP status after lifestyle modification [86]. Intermediate-risk and high-risk patients with stage 1 hypertension require immediate antihypertensive medications [87]. Because most randomized clinical trials and meta-analyses show that antihypertensive medications are effective in reducing CV risk, antihypertensive medications along with lifestyle modification is recommended in stage 2 hypertension [85, 87].

**Table 8:**
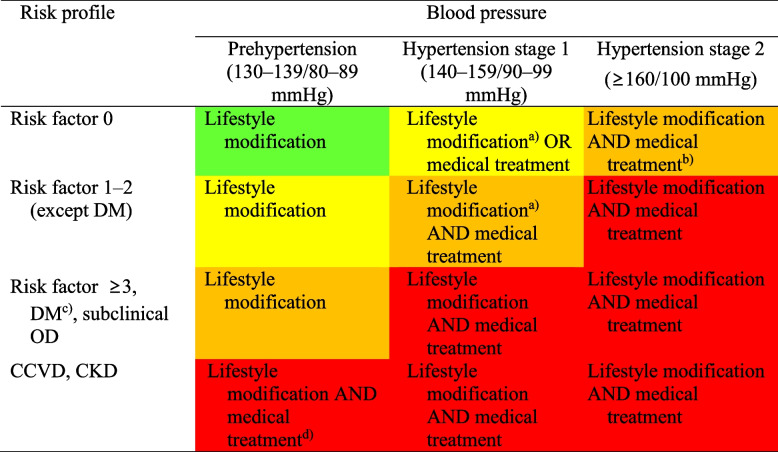
Cardiovascular risk and treatment strategy for hypertension

The colors indicate 10-year cardiocerebrovascular event rate: green, < 5%; yellow, low risk (5–10%); orange, moderate risk (10–15%); and red, high risk (> 15%)

*DM* diabetes mellitus, *OD* organ damage, *CCVD* cardiocerebrovascular disease, *CKD* chronic kidney disease

^a)^Life style modification within 3 months; ^b)^Immediate medical treatment can be started according to blood pressure level; ^c)^With hypertension and one or more other risk factors; ^d)^Medical treatment for combined cardiovascular or renal diseases

### Target blood pressure

**Table:** 

Recommendation	Class	Level	Reference
It is recommended to control BP to less than 140/90 mmHg in low-risk and intermediate-risk groups.	I	A	[86, 88]
It is reasonable to reduce BP below 130/80 mmHg in patients with coronary artery disease (CAD), peripheral artery disease, abdominal aortic aneurysm, heart failure, and left ventricular hypertrophy.	IIa	B	[58]
It is reasonable to reduce BP below 130/80 mmHg in individuals with high CV risk.^a)^	IIa	B	[89, 90]

^a)^For detailed information on target BP in the elderly (≥65 years) and CKD, refer to each corresponding section

**Fig. 2:**
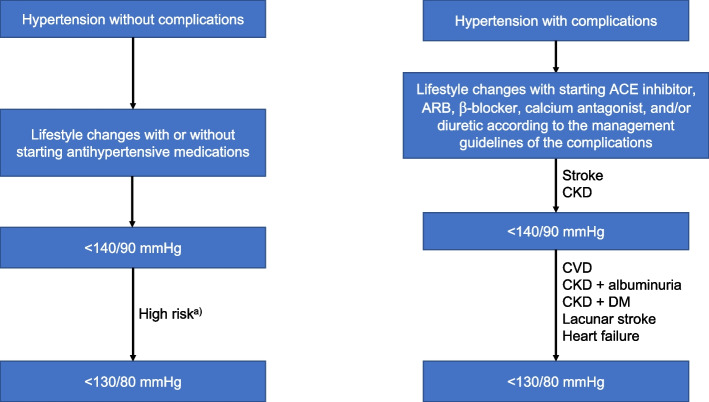
Clinical algorithms for reaching target blood pressure. ACE, angiotensin converting enzyme; ARB, angiotensin receptor blocker; CVD, cardiovascular disease; CKD, chronic kidney disease; DM, diabetes mellitus. ^a)^Subclinical organ damage, cardiovascular risk factors ≥3, DM with cardiovascular risk factors ≥1

In general, the target SBP is < 140 mmHg and target DBP is < 90 mmHg unless in clinical conditions shown in Table 9 [85, 91, 92]. It is reasonable to reduce BP below 130/80 mmHg in individuals with CVD or high CV risk [58, 89]. On the other hand, it is recommended to control BP to less than 140/90 mmHg in low-risk and intermediate-risk groups [88]. Clinical algorithms for reaching target BP are shown in Fig. 2. The main rationale of changing target BPs from around 130/80 mmHg to below 130/80 mmHg in high-risk patients is the adoption of corresponding BPs, which emphasize the balanced awareness of both WCE and masked effects during intensive BP control. Some studies for corresponding BPs using AOBP also support the idea that too much concern about WCE may result in undertreatment within the intensive target BP range. According to these corresponding BPs, achieved daytime ambulatory SBP in the intensive arm in Systolic Blood Pressure Intervention Trial (SPRINT), i.e., 126 mmHg could be equivalently applied to conventional OBP during which OBP is complemented by HBPM.

**Table 9:** Target BP in hypertension treatment

Clinical situation	SBP (mmHg)	DBP (mmHg)	COR	LOE
Hypertension without complications				
Low to intermediate cardiovascular risk	<140	< 90	I	A
Elderly	<140	< 90	I	A
High cardiovascular risk^a)^	< 130	< 80	IIa	B
Diabetes mellitus	< 130	< 80	IIa	B
Low to intermediate risk	< 140	< 90	I	A
High risk^b)^	< 130	< 80	IIa	B
Hypertension with complications				
Cardiovascular disease^c)^	< 130	< 80	IIa	B
Chronic kidney disease				
Without albuminuria^d)^	<140	< 90	I	A
With albuminuria	< 130	< 80	IIa	B
With diabetes mellitus	< 130	< 80	IIa	B
Stroke	<140	< 90	I	B
Lacunar stroke	< 130	< 80	IIa	B

*BP* blood pressure, *SBP* systolic blood pressure, *DBP* diastolic blood pressure, *COR* class of recommendation, *LOE* level of evidence

^a)^Subclinical organ damage or cardiovascular risk factors ≥3; ^b)^Subclinical organ damage or cardiovascular risk factors ≥1; ^c)^Coronary artery disease, peripheral artery disease, abdominal aortic aneurysm, heart failure, or left ventricular hypertrophy; ^d)^Microalbuminuria or macroalbuminuria

### Antiplatelet therapy

**Table:** 

Recommendation	Class	Level	Reference
It is recommended to use aspirin in hypertensive patients with CVD.	I	A	[93]
Low-dose aspirin for primary prevention may be considered in high-risk hypertensive individuals aged 40 to 70 years without CVD.	IIb	B	[94, 95]
The use of aspirin for primary prevention is not recommended in hypertensive patients over 70 years of age with low or intermediate CV risk.	III	A	[78, 94–96]

It is clear that antiplatelet therapy can be used for secondary prevention after the onset of CVD in hypertensive patients [93]. However, the study results are inconsistent in terms of the effect of antiplatelet therapy in the primary prevention of CVD. Although some guidelines recommended not using aspirin for primary prevention [78, 96], use of low-dose aspirin still is beneficial for primary prevention in some patients with high-risk profiles [94, 95]. To reduce CV risk, antiplatelet agents, such as low-dose aspirin (100 mg), can be used to hypertensive individuals aged 40 to 70 years with high CV risk [93–95]. It is a general recommendation in most practice guidelines to discourage the use of aspirin for primary prevention in the older patients, patients at high bleeding risk and patients low or intermediate CV risk [78, 94–96]. Antiplatelet agents are administered after BP is controlled, while bleeding complications are frequently checked.

### Antidyslipidemic therapy

**Table:** 

Recommendation	Class	Level	Reference
In hypertensive patients with intermediate or high CV risk, statin is recommended.	I	A	[97, 98]
In hypertensive patients with CVD, statin is recommended.	I	A	[99]
It is recommended that low-density lipoprotein (LDL) cholesterol level should be reduced to < 70 mg/dL in hypertensive patients with CVD.	I	A	[99]

Lipid-lowering drug therapy is effective in preventing CVD in high-risk hypertensive patients. In hypertensive patients without CVD, statin was used to lower the LDL-cholesterol level by > 50%, when LDL-cholesterol was ≥130 mg/dL, and its CVD prevention effect is evident [97]. It is recommended to lower LDL-cholesterol to less than 70 mg/dL in hypertensive patients with CVD [99].

### Blood glucose control

**Table:** 

Recommendation	Class	Level	Reference
If there is no risk of hypoglycemia in diabetic patients with hypertension, it is recommended to lower glycated hemoglobin to < 6.5%.	I	A	[100]

The goal of glycemic control in diabetic patients is less than 6.5% of glycated hemoglobin. The glycated hemoglobin target may be lowered either if disease duration of diabetes is shorter or if there are no accompanying complications and low risk of hypoglycemia. However, glycemic control goals can be individualized in patients with severe hypoglycemia, short life expectancy, advanced microvascular and macrovascular complications, and the risk of developing hypoglycemia in the elderly aged 75 years or older [100]. Since sodium glucose cotransporter-2 (SGLT-2) inhibitors not only lower BP, but also reduces the occurrence of CVD and slows the deterioration of kidney function, use of these drugs should be considered in hypertensive patients with diabetes [101, 102]. When used together with loop diuretics, caution should be paid because as excessive loss of body fluid may occur due to increased urine output.

### Patient monitoring and follow-up

**Table:** 

Recommendation	Class	Level	Reference
For patients taking antihypertensive drugs, follow-up and monitoring using home BP is recommended.	I	A	[103, 104]

When starting a new antihypertensive drug or adjusting its dosage or dosing time, it is recommended to follow-up at least monthly until the target BP is reached to check the adherence to the drug and whether the BP is controlled [105, 106]. Stage II or more severe hypertension may be followed up more frequently. Electrolyte and kidney function tests are performed at least one to two times a year [107]. If the target BP is reached and remains stable, patients are followed up every 3 to 6 months. Check whether patients’ adherence to drug therapy decreases with increasing follow-up intervals. Care should be taken to avoid missing blood tests. Encouragement to measure home BP can help determine BP control when follow-up intervals are extended [103, 104].

### Adherence

**Table:** 

Recommendation	Class	Level	Reference
As reduced dosing frequency is associated with better adherence, antihypertensive drugs are recommended to be administered once a day unless there are special situations such as resistant hypertension, morning hypertension, medication adjustment.	I	A	[108, 109]
In stable patients with the same drug and dosage during a long period of time, it is reasonable to use fixed-dose combination drugs because their drug adherence is better than that of free combination drugs.	IIa	B	[110, 111]
The use of the fixed-dose combination of antihypertensive drugs and statins to increase adherence to drug therapy may be considered.	IIb	B	[112]

Reducing the number of dosing increases patient’s adherence, so once-daily antihypertensive dosing is recommended unless there is a special reason [109]. In patients who are stably being taking the same drug and dosage during a long period of time, the administration of fixed-dose combination drug is considered because it has better adherence than combination therapy of free drugs [110, 111, 113]. About half of hypertensive patients have dyslipidemia, so the number of patients taking both antihypertensive medications and statins is increasing. The use of the fixed- dose combination of antihypertensive drugs (especially calcium channel blockers and angiotensin receptor blocker [ARB]) and statins also improves patient adherence compared to free combination [112, 114].

## Hypertension in special situations

### Diabetes mellitus

**Table:** 

Recommendation	Class	Level	Reference
In diabetic patients without CV risk factors, clinical CVD, stages 3, 4, or 5 CKD, and subclinical organ damage, it is recommended to control BP below 140/90 mmHg.	I	A	[92, 115–118]
In diabetic patients with CV risk factors ≥1, CVD, stages 3, 4, or 5 CKD, and subclinical organ damage, it is reasonable to control BP below 130/80 mmHg.	IIa	B	[119, 120]
In hypertensive patients with DM, all five classes of antihypertensive drugs can be recommended as first-line drugs.	I	A	[118, 121]
Angiotensin converting enzyme (ACE) inhibitors or ARBs are recommended if microalbuminuria or proteinuria is present.	I	B	[122–124]

Evidence for the drug treatment of prehypertension in diabetic patients is still limited. In diabetic patients, lowering BP below 130/80 mmHg did not prove a preventive effect on CVD. Rather, lowering BP to < 130/80 mmHg worsened renal function in diabetic patients. Therefore, a general target BP of less than 140/90 mmHg is recommended for hypertensive patients with DM [92, 115–118]. However, in diabetic patients with high-risk clinical features including CV risk factors ≥1, CVD, stages 3, 4, or 5 CKD, and subclinical organ damage, it is considered to control BP below 130/80 mmHg [119, 120].

Among oral antidiabetic drugs, SGLT-2 inhibitors have an antihypertensive effect [125]. Thus, it may be necessary to adjust the dosage of antihypertensive drugs when used with SGLT-2 inhibitors.

### Hypertension in the elderly

**Table:** 

Recommendation	Class	Level	Reference
SBP goal below 140 mmHg is recommended for noninstitutionalized ambulatory community-dwelling adults (≥65 years of age).	I	A	[58, 119]

Although results of SPRINT [58] and the Strategy of Blood Pressure Intervention in the Elderly Hypertensive Patients (STEP) trial [119] have shown that intensive BP-lowering therapy is effective in elderly hypertensive patients, additional research is needed on target BP in adults at very old age, the frail elderly, and the elderly in a facility.

Orthostatic BP should be periodically measured to check for orthostatic hypotension. Since intensive BP control rather reduces the risk of orthostatic hypotension, there is no need to reduce the drug if orthostatic hypotension is suspected [126]. Treatment of orthostatic hypotension includes nondrug treatment such as water/salt intake, cross-legged and squatting postures, use of compression stockings, and treatment with drugs such as midodrine, pyridostigmine and fludrocortisone [127].

### Cardiovascular disease

#### Coronary artery disease

**Table:** 

Recommendation	Class	Level	Reference
It is reasonable to keep BP below 130/80 mmHg in hypertensive patients with CAD.	IIa	B	[58, 128–130]

Considering the “J curve” phenomenon in hypertensive patients with CAD, an excessive decrease in SBP increases CV risk, which is more pronounced in patients with left ventricular hypertrophy. Therefore, hypertensive patients with CAD should be carefully managed so as not to lower SBP to less than 110 mmHg and DBP to less than 70 mmHg [131].

As antihypertensive medication, β-blockers should be considered first for 1 day to 1 month after acute myocardial infarction [87]. ACE inhibitors are also effective in patients with acute myocardial infarction [132]. In hypertensive patients with CAD, β-blockers, ACE inhibitors, or ARBs are recommended as first-line drugs. When symptom or BP control is not sufficient with these drugs, calcium channel blockers, diuretics, and aldosterone antagonists are recommended [87, 129, 130, 133].

#### Atrial fibrillation

**Table:** 

Recommendation	Class	Level	Reference
The use of ARBs in hypertensive patients with atrial fibrillation is reasonable because it reduces the recurrence of atrial fibrillation.	IIa	B	[134, 135]

If hypertensive patients have atrial fibrillation, the risk of thromboembolism is high, so anticoagulation is considered unless contraindicated [136, 137].

#### Aortic disease

Coarctation of aorta is usually corrected with surgical treatment in childhood. However, even after surgical correction, hypertension can be developed in childhood, requiring long-term follow-up. In some patients, coarctation of aorta is not found until adulthood, which progresses to severe hypertension, resulting in cardiac hypertrophy, left ventricular hypertrophy, and target organ damage accompanied by extensive collateral circulation down the coarctation site. However, there is still randomized study on an appropriate BP treatment strategy in patients with coarctation of aorta [138].

Bicuspid aortic valve is common in men and related to coarctation of aorta. Also, patients with bicuspid aortic valve are more likely to develop aortic disease than the general population. If BP is not controlled, it can progress to aortic aneurysm, leading to dangerous consequences such as aortic rupture [139, 140]. Therefore, it may be helpful to control BP to be below 130/80 mmHg in patients with bicuspid aortic valve. ARBs can be used when BP control is required in patients with severe aortic stenosis [141].

### Chronic kidney disease

**Table:** 

Recommendation	Class	Level	Reference
For CKD patients with hypertension, a target BP below 140/90 mmHg is recommended.	I	A	[115, 142, 143]
For CKD patients with hypertension and albuminuria, a target BP below 130/80 mmHg is reasonable.	IIa	B	[144, 145]
In hypertensive patients with CKD, combination therapy with ACE inhibitors, ARBs, or direct renin inhibitors are not recommended.	III	B	[146–149]

The main aim of controlling BP in patients with CKD is to slow the deterioration of renal function and reduce the morbidity and mortality of CVD. The SPRINT study has shown benefits in all-cause mortality and cardiovascular morbidity with a target SBP of < 120 mmHg in the hypertensive CKD subgroup without diabetes [58, 150]. However, the evidence for the benefit of a target SBP of less than 120 mmHg is less convincing in CKD at age < 50 years, diabetes, advanced CKD stage (eGFR < 20 mL/min/1.73m^2^), proteinuria > 1 g/day, and polycystic kidney disease [151]. The updated Kidney Disease: Improving Global Outcomes (KDIGO) 2021 Guideline for the management of BP in CKD patients who have not receive dialysis is based on the results of the SPRINT study, which used a standardized OBP measurement as a principle method. Because of difficulties in using a standardized OBP measurement in clinical practice, there are limitations in applying the updated target BP for CKD patients [148].

In randomized controlled trials and a large network meta-analysis, combination therapy was compared to monotherapy. Despite lowering proteinuria in the short term, combination therapy did not reduce all-cause mortality or cardiovascular morbidity. Also, it did not slow the progression of CKD to end-stage renal disease; however, combination therapy increased the incidence of acute renal injury and hyperkalemia compared to monotherapy [146–148]. These results were similar in combination therapy with ARBs and direct renin inhibitors [149].

### Cerebrovascular disease

#### Blood pressure control in acute ischemic stroke

**Table:** 

Recommendation	Class	Level	Reference
In patients who are treated with intravenous recombinant tissue plasminogen activator (IV-TPA), in order to reduce the risk of intracerebral hemorrhage (ICH), it is reasonable to lower BP to < 185/100 mmHg before treatment and to maintain BP < 180/105 mmHg during the first 24 hours.	I	B	[152, 153]
For patients undergoing endovascular recanalization therapy (ERT), it is reasonable to maintain preoperative BP < 185/110 mmHg to reduce the risk of cerebral hemorrhage. During the first 24 hours after ERT, the optimal BP level remains uncertain and should be individualized based on the patient’s clinical and imaging profiles. In general, maintaining BP < 180/105 mmHg may be considered. However, a lower BP level may be considered in patients who achieved successful reperfusion.	IIb	C	[154–161]
In patients with persistent high BP levels of > 140/90 mmHg and in a stable neurological condition without contraindications to BP lowering, it is reasonable to initiate antihypertensive therapy before or at discharge in order to improve long-term BP control.	IIa	B	[162, 163]
The benefit of BP lowering within 48 to 72 hours after stroke onset is uncertain in acute ischemic stroke patients with BP ≥220/120 mmHg not receiving IV-TPA or ERT and having no comorbidities. If BP lowering is required based on clinical judgment, BP lowering by approximately 15% may be considered during the first 24 hours.	IIb	C	[7]
In acute ischemic stroke patients with BP < 220/120 mmHg not receiving IV-TPA or ERT and having no comorbidities, initiating BP lowering within 48 to 72 hours after stroke onset is not recommended because it neither improves functional disabilities nor reduces major vascular events at 3 to 6 months.	III	A	[162–164]

#### Acute parenchymal hemorrhage

**Table:** 

Recommendation	Class	Level	Reference
In patients with acute ICH presenting within 6 hours of the onset who have an SBP level between 150 and 220 mmHg, rapid lowering of SBP to 140 mmHg may be considered. However, excessive BP lowering (SBP < 140 mmHg) is not usually recommended because it does not have additional benefit on functional outcome and potentially increases the risk of renal dysfunction.	IIb	A	[165, 166]
In patients with acute ICH who have an elevated SBP > 220 mmHg, it is reasonable to reduce BP with intravenous antihypertensive agent infusion along with close BP monitoring.	IIa	C	[167–169]

#### Secondary prevention

**Table:** 

Recommendation	Class	Level	Reference
In patients with stroke or transient ischemic attack (TIA) who have previously or newly been diagnosed with hypertension of an established BP of ≥140/90 mmHg, antihypertensive treatment should be restarted or initiated several days after the stroke or TIA to reduce the risk of recurrent stroke and other CV events.	I	A	[170–172]
Treatment with thiazide diuretics, ACE inhibitors, or ARBs, or combination treatment consisting of thiazide diuretics plus ACE inhibitors, is recommended for adults who experience a stroke or TIA.	I	A	[170, 172, 173]
It is reasonable to consider using calcium blockers in order to control hypertension in patients with stroke or TIA.	IIa	C	[174]
For adults with a lacunar stroke, a target SBP goal of less than 130 mmHg is reasonable.	IIa	B	[175]

### Erectile dysfunction

The relationship of hypertension and antihypertensive drugs with sexual dysfunction in women is not so clear as in men [176]. According to the SPRINT study, hypertension and use of antihypertensive drugs were not associated with sexual dysfunction in middle-aged and elderly women [177].

In order to increase adherence to antihypertensive medications, all hypertensive patients should be regularly checked for sexual dysfunction during the follow-up period as well as at the early stage of diagnosis. In particular, for men complaining of sexual dysfunction, it is recommended to avoid prescription of β-blockers or diuretics, which are relatively related to sexual dysfunction, or to substitute other drugs for them.

### Pregnancy

Hydralazine, methyldopa, labetalol, and nifedipine are useful oral antihypertensive drugs during pregnancy [178]. However, methyldopa is not available in Korea. In emergency situations such as preeclampsia, intravenous labetalol is recommended; however, intravenous nitroprusside or nitroglycerin and oral nifedipine can also be used.

### Cognitive impairment

**Table:** 

Recommendation	Class	Level	Reference
Hypertension treatment to prevent cognitive dysfunction and dementia in adult hypertensive patients is reasonable.	IIa	A	[179–186]

Although previous studies have not shown a statistically significant preventive effect due to problems such as lack of power and short follow-up duration, it has been found that active BP-lowering treatment tends to reduce the incidence of dementia [179–186].

## Data Availability

Not applicable.

## Abbreviations

ABPM: Ambulatory blood pressure monitoring
ACE: Angiotensin converting enzyme
ACR: Albumin to creatinine ratio
AOBP: Unattended automated office blood pressure
ARB: Angiotensin receptor blocker
BP: Blood pressure
CAD: Coronary artery disease
CKD: Chronic kidney disease
COR: Class of recommendation
CV: Cardiovascular
CVD: Cardiovascular disease
DBP: Diastolic blood pressure
DM: Diabetes mellitus
eGFR: Estimated glomerular filtration rate
ERT: Endovascular recanalization therapy
HBPM: Home blood pressure monitoring
HMOD: Hypertension-mediated organ damage
ISO: International Organization for Standardization
ICH: Intracerebral hemorrhage
IV-TPA: Intravenous recombinant tissue plasminogen activator
KDIGO: Kidney Disease: Improving Global Outcomes
Kor-ABP: Korean Ambulatory Blood Pressure Monitoring
LDL: Low-density lipoprotein
LOE: Level of evidence
MH: Masked hypertension
MUCH: Masked uncontrolled hypertension
OBP: Office blood pressure
SBP: Systolic blood pressure
SGLT-2: Sodium glucose cotransporter-2
SPRINT: Systolic Blood Pressure Intervention Trial
TIA: Transient ischemic attack
STEP: Strategy of Blood Pressure Intervention in the Elderly Hypertensive Patients
WCE: White coat effect
WCH: White coat hypertension
WCUH: White coat uncontrolled hypertension
